# Atrial Functional Tricuspid Regurgitation Associated With Atrial Standstill

**DOI:** 10.7759/cureus.69577

**Published:** 2024-09-17

**Authors:** Hidetsugu Hori, Tomokazu Kosuga, Yukio Hosokawa, Keiichiro Tayama, Kenichi Kosuga

**Affiliations:** 1 Cardiothoracic Surgery, Munakata Suikokai General Hospital, Fukutsu, JPN

**Keywords:** atrial fibrillation, atrial standstill, functional tricuspid regurgitation, tricuspid regurgitation, tricuspid valve replacement

## Abstract

We report a case of atrial functional tricuspid regurgitation with an atrial standstill in a 71-year-old woman with a history of chronic atrial fibrillation (AF). The ECG showed a flat baseline with no AF waves and regular, narrow QRS complexes, whereas the previous ECG demonstrated AF. Echocardiography revealed dilation of the right atrium and the tricuspid annulus with severe regurgitation, but mitral regurgitation was mild. No atrial contraction was detected. Bilateral ventricular function was preserved. Cardiac catheterization showed no pulmonary hypertension and an a-wave in atrial pressure tracings. During surgery, epicardial pacing was unable to capture both atria. The tricuspid valve was replaced and a pacemaker was implanted.

## Introduction

Atrial fibrillation (AF) leads to the enlargement of both atria, which results in annular dilation of both the mitral and tricuspid valves, causing valvular regurgitation. This annular dilation and valvular regurgitation are significantly more pronounced in the tricuspid valve (TV) than in the mitral valve [1]. Significant (≥ moderate) tricuspid regurgitation (TR), without other associated cardiac abnormalities, was found in 25% of patients with chronic AF lasting more than 10 years [2]. This pathology is termed atrial functional TR (AFTR). Although AFTR is relatively frequently experienced in clinical settings, AFTR associated with atrial standstill (AS) has been reported in only a few patients [3]. In this paper, we report a case of severe AFTR associated with AS, resulting from chronic AF in an elderly patient.

## Case presentation

**Figure 1:**
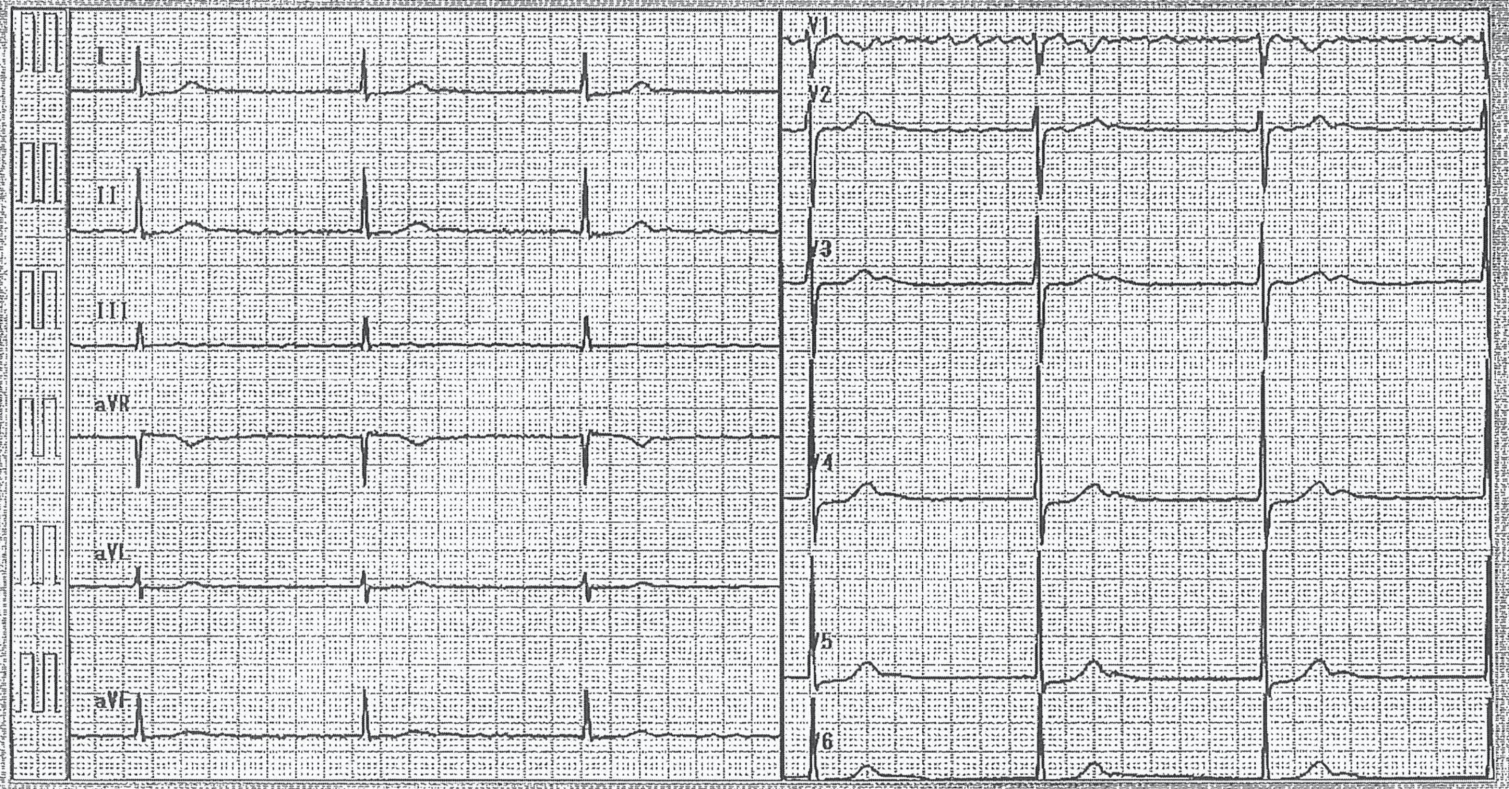
A previous ECG showing AF with a slow ventricular response. AF: Atrial fibrillation

A 71-year-old woman was referred for treatment of right heart failure (RHF) due to severe TR. The patient had a history of untreated AF at the age of 55 and cerebral embolism at age 62. At the age of 62, she was also diagnosed with AF and a slow ventricular response on ECG (Figure 1).

**Figure 2:**
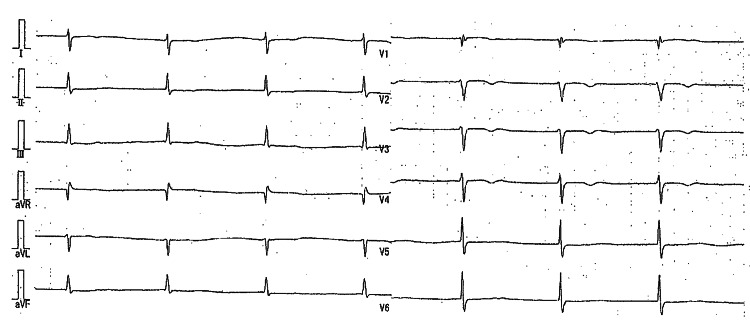
An ECG on admission revealed AS with a junctional escape rhythm. AS: Atrial standstill

Transthoracic echocardiography (TTE) showed enlargement of both atria, and the left ventricular ejection fraction (EF) was 78%. The Doppler study revealed moderate tricuspid regurgitation and mild mitral regurgitation. Over the next 10 years, the patient was hospitalized twice for RHF. Upon admission, she was edematous. Her blood pressure was 108/58 mmHg, and her pulse rate was 45/min. A systolic murmur was heard at the apex. The liver was palpable 5 cm below the right costal margin. The hematological examination revealed a hemoglobin level of 8.6 g/dl, a creatinine level of 1.25 mg/dl, and an estimated glomerular filtration rate (eGFR) of 33.0 ml/min. The chest X-ray indicated cardiomegaly (cardiothoracic ratio (CTR) 74%) with no signs of pulmonary congestion. The ECG revealed a flattened baseline without P-waves or f-waves, regular and narrow QRS complexes, and a heart rate of 39/min, suggesting AS with a junctional escape rhythm (Figure 2).

**Figure 3:**
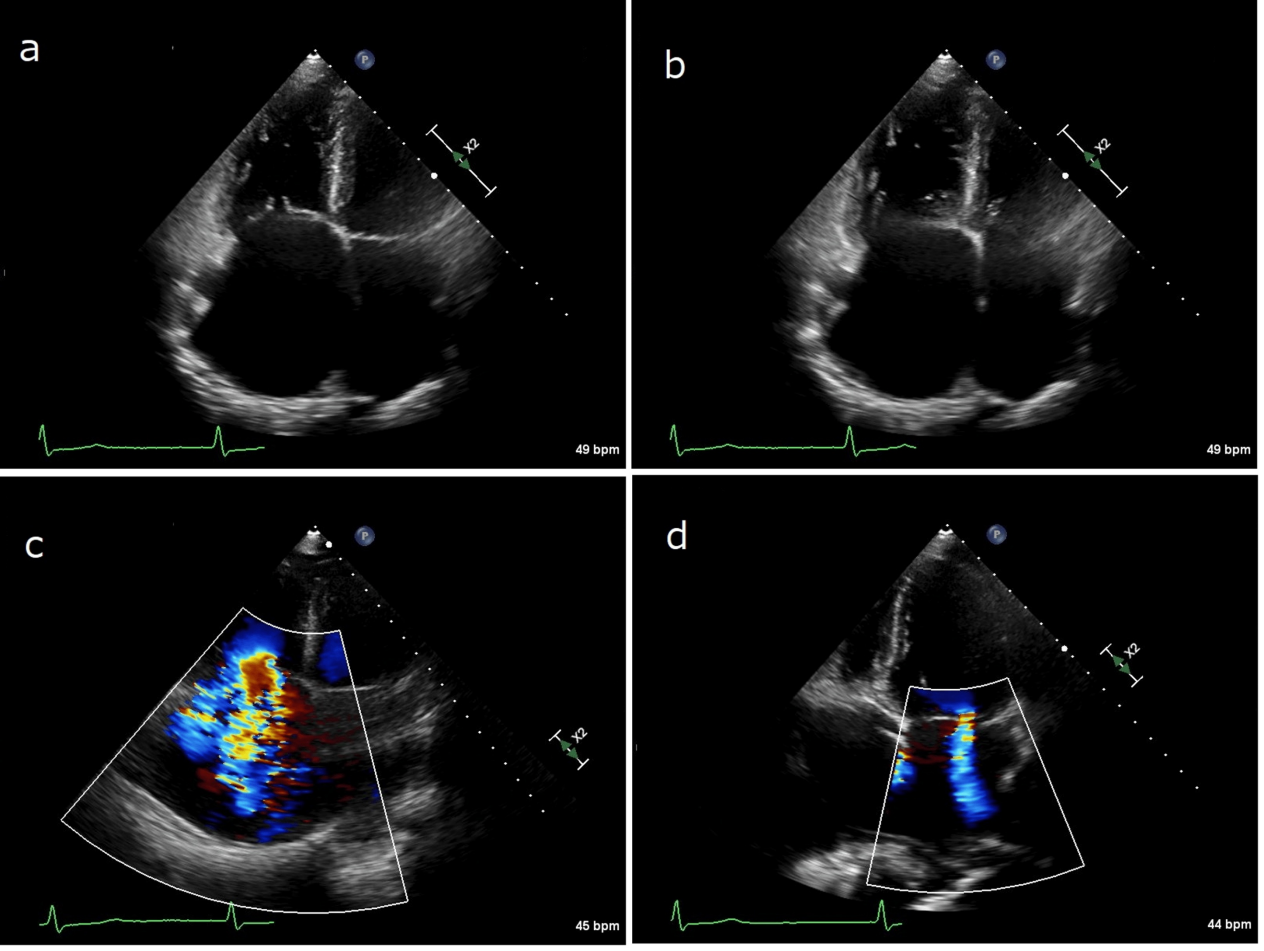
TTE on admission a: Systolic function; b: Diastolic function. Both TTEs (a and b) showed dilation of both atria, particularly the RA, and a markedly dilated tricuspid annulus with loss of coaptation of the valve leaflets; atrial contraction was absent. c: A Doppler study on admission showed severe TR; d: A Doppler study on admission showed trivial MR TTE: Transthoracic echocardiography, RA: Right atrium, TR: Tricuspid regurgitation, MR: Mitral regurgitation

The TTE demonstrated enlargement of both atria, particularly the right atrium (RA), with the RA area measuring 52.3 cm2, and the right ventricle (RV). The markedly dilated TV annulus (4.6 cm) and loss of coaptation between the anterior and septal tricuspid leaflets were also detected, but no structural abnormalities were found in the TV or the mitral valve (Figure 3 a and b).

In addition to the absence of A-waves on the mitral valve tracing, no contraction was observed in either atrium and abnormal wall motion was not detected in either ventricle. The fractional area change of the RV and the left ventricular EF were 34.2% and 68.0%, respectively. The dilated inferior vena cava (2.8 cm) did not collapse with inspiration. The Doppler study revealed severe TR (effective regurgitant orifice area 41 mm^2^; Figure 3 c) with systolic flow reversal in the hepatic veins and mild mitral regurgitation (Figure 3 d). No intracardiac thrombi or shunts were present. Cardiac catheterization demonstrated a pulmonary artery pressure of 43/13 mmHg, mean pulmonary capillary wedge pressure of 14 mmHg, and mean RA pressure of 16 mmHg, but no A-wave was found in the RA or pulmonary capillary wedge pressure tracings. Coronary angiography showed normal arteries.

During surgery, no atrial contraction or fibrillation was observed macroscopically. Temporary epicardial pacing was attempted at multiple sites on both atria using the maximum pulse amplitude, but it failed to capture both atria. Both ventricles were easily paced at low pulse amplitude, but no retrograde atrial activity was observed during ventricular pacing. After establishing cardiopulmonary bypass and inducing cardiac arrest, the RA was opened. Remarkable annular dilatation of the TV without pathologic lesions was confirmed. Tricuspid valve replacement (TVR) with a bioprosthetic valve and epicardial closure of the left atrial appendage using a clip were performed. Finally, a permanent pacemaker was implanted in the RV wall. Cardiopulmonary bypass was discontinued with ventricular pacing. The latest TTE showed normal prosthetic valve function with trivial transvalvular regurgitation, mild mitral regurgitation, and normal left ventricular function (EF 67%). The patient has controlled RHF five years postoperation.

## Discussion

This case demonstrates that significant AFTR leading to RHF may necessitate early therapeutic intervention and that chronic AF causing significant TR can eventually progress to AS, which further exacerbates RHF due to bradycardia. It is well-recognized that significant AFTR is commonly found in elderly people, and it increases with age. However, the natural history of AFTR is not fully understood. Topilsky et al. [4] showed a poor prognosis for severe AFTR (five-year survival rate and cardiac event-free rate of 60% and 58%, respectively). They have emphasized that an effective regurgitant orifice area (≥40 mm^2^) is a strong predictor of lower survival and higher cardiac event rates. However, a follow-up study of Japanese patients indicated that the mortality rate caused by RHF was low, with a survival rate and cardiac event-free rate at five years of 78% and 73%, respectively. However, the prognosis was poor for patients with persistent TR who had previously been hospitalized due to RHF, with a 26% incidence of cardiac death within five years post-hospitalization [5]. The study identified large RV outflow tract dimensions (>35.3 mm), RA area (>40.3 cm^2^), and tenting height of the TV (>2.1 mm) on TTE as strong predictors of hospitalization due to RHF, in addition to decreased eGFR levels [5]. The study has emphasized that therapeutic intervention should be considered for patients with persistent TR who have been hospitalized due to RHF, even if symptoms and signs of RHF improve after medical treatment. It also suggests that early intervention should be considered for those who exhibit these predictors [5].

However, Axtell et al. [6] recently demonstrated that isolated TV surgery did not improve long-term survival compared to medical treatment, and no survival difference was observed between TV repair and TVR. Regarding the lack of survival advantage with surgery, it has been suggested that surgical mortality could be negatively impacted by delayed referral for TV surgery, which allows the development of overt RHF or end-organ damage [6]. A nationwide study in the US showed that patients undergoing TV surgery were often referred late, as evidenced by the high prevalence of advanced liver disease, acute decompensated congestive heart failure, non-elective surgical status, and significant rates of unplanned readmissions prior to surgery. These factors were the strongest predictors of mortality following isolated TV surgery. [7] These findings suggest that an isolated TV operation itself may not inherently carry a high risk if performed in a timely manner, despite previous documentation of high operative mortality. Tricuspid regurgitation must be acknowledged as a severe disease that reduces survival, limits functional capacity, and leads to end-organ dysfunction. Appropriate referral for early surgical intervention is necessary when medical treatment for TR proves ineffective.

Atrial standstill was first described in 1946 by Chavez et al. [8]. Atrial fibrillation is the most common type of arrhythmia and can lead to devastating complications such as stroke [9] and heart failure [10] if not treated. Long-standing AF may cause AS [11]. The diagnosis of AS is confirmed by both electrical and mechanical silence of the atria, and its diagnostic criteria are defined as follows: (1) absence of P-waves on surface and intracardiac ECGs; (2) absence of A-waves in jugular venous pulse and right atrial pressure tracings; (3) supraventricular type QRS complex; (4) immobility of the atria on fluoroscopy and/or angiography; and (5) inability to electrically stimulate the atria [12]. Our patient met all the criteria except for recording an intracardiac ECG.

Although the duration of AS was unknown in our patient, AS, likely persistent, subsequently developed due to chronic AF. In fact, a progressive reduction in AF waves that eventually disappeared from all ECG leads was confirmed in a few patients with severe AFTR and AS [2]. In a review study of patients with AS [13], the AS developed progressively as a sequela to long-standing cardiac disease with a prior history of supraventricular arrhythmias in about one-third of the patients. Pathological studies demonstrated severe and widespread atrial fibrosis and degeneration. With the progression of dilation of the TV annulus and worsening of the TR, the degenerative lesions that have extended to both atria over the last 10 years may have eventually advanced chronic AF to AS in our patient.

## Conclusions

We report a case of AFTR associated with AS resulting from chronic AF in the elderly, which was managed by TVR with a bioprosthetic valve and pacemaker implantation. Atrial fibrillation develops enlargement of both atria, which leads to annular dilatation of both mitral and tricuspid valves, resulting in valvular regurgitation. This pathology is termed AFTR. With the progression of dilatation of the TV annulus and worsening of the TR, the degenerative lesions extended to both atria may have eventually advanced chronic AF to AS. This case demonstrates that significant AFTR leading to RHF may require early therapeutic intervention and that chronic AF causing significant TR may eventually progress to AS, which induces further deterioration of RHF due to bradycardia.
